# Anorectal malformation combined with Hirschsprung's disease: a case report

**DOI:** 10.3389/fped.2023.1182342

**Published:** 2023-05-24

**Authors:** Jiansen Fan, Mingkun Liu, Yu Lin, Yuanbin He, Yifan Fang

**Affiliations:** Fujian Children's Hospital (Fujian Branch of Shanghai Children's Medical Center), College of Clinical Medicine for Obstetrics & Gynecology and Pediatrics, Fujian Medical University, Fuzhou, China

**Keywords:** anorectal malformation, Hirschsprung's disease, barium enema examination, enterocolitis, severe constipation

## Abstract

Anorectal malformation (ARM) and Hirschsprungs disease (HSCR) are frequently associated with other congenital malformations, but rarely with one another. We describe the case of a child with intermediate anorectal malformation who underwent ARM correction. This child experienced recurrent postoperative symptoms, including intestinal obstruction, nutrition intolerance, and weight loss. The child was diagnosed with Hirschsprung's disease by colon barium contrast and pathological findings from a rectal biopsy, and subsequently underwent pull -through procedure after conservative treatment failed. After six months of postoperative follow-up, the patient still experiences occasional episodes of enteritis, but the symptoms are substantially less severe than they were before surgery, and the patient's weight is slowly increasing. We described a case of a child who had ARM combined with HSCR. Although the association between ARM and HSCR is uncommon, severe constipation or enteritis following complete correction of ARM in the absence of anal stricture should prompt consideration for HSCR. Before the second stage of ARM surgery, pay close attention to the barium enema examination, as an abnormal shape may indicate the presence of HSCR.

## Introduction

Anorectal malformation (ARM) is a complex congenital rectal disease of anus, rectum and urogenital system.With an incidence of 1/5,000 to 1/1,500, it is the most prevalent malformation of the neonatal digestive system. Hirschsprung's disease (HSCR) is incredibly rare, and some researchers have estimated that its prevalence ranges from 1/250,000 to 1/75,000 (1–3). Constipation is the most commonly postoperative complication of ARM. As a result, it is commonly mistaken for the two, which can result in delayed treatment, adverse effects on children’s prognoses, or even child fatalities (4, 5). We described a case of a child who had ARM combined with HSCR, offering doctors a point of reference.

## Case description

A male (G2P2, 40 + 3 weeks, vaginal birth, 3,700 g birth weight) was admitted to our facility due to the absence of an anus in the anal fossa. Neither parent had a family history of digestive or liver disease. There was no substance misuse by the mother during pregnancy. Prenatal examination did not reveal any abnormalities.

**Figure 1:**
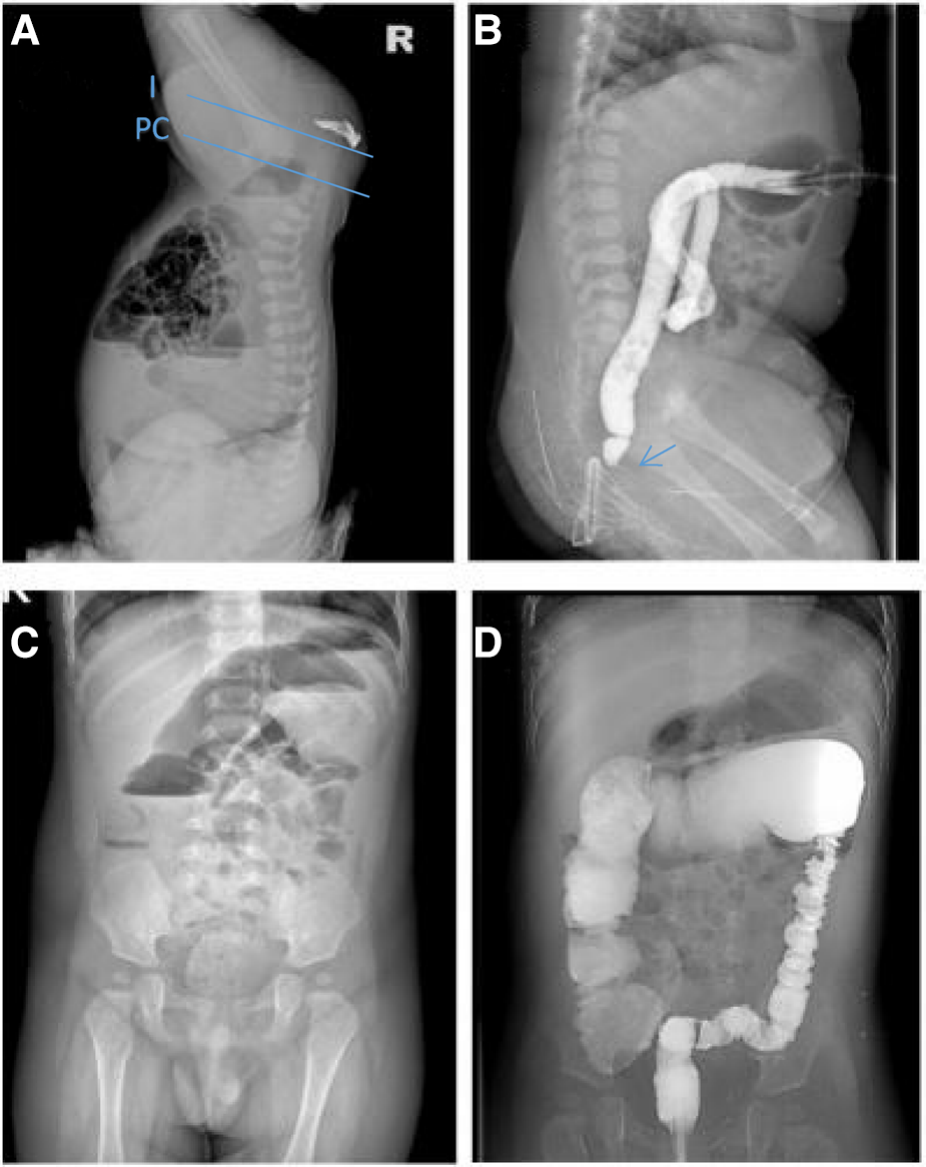
(**A**) abdominal x-rays before the first surgery. (**B**) before the second surgery. (**C,D**) after postoperative correction of anal atresia.

Twenty-four hours after birth, an inverted abdominal radiograph revealed that the rectum was approximately 1.8 cm away from the marker of the anal opening in dimple, Blind rectal end between lines PC and I (Figure 1A). He was diagnosed with intermediate anal atresia and underwent double transverse colostomy. On the third day following surgery, he had fully recovered and was discharged. When he returned to the hospital for treatment at the age of 3 months, a barium enema examination revealed that the distal colon was small and the colon pocket was not evident, the end of the rectum was connected to the urethra, and a fistula shadow, approximately 0.4 cm wide and 0.2 cm long, was visible in the middle (Figure 1B). The patient was diagnosed with an ARM-type rectoprostatic fistula and underwent laparoscopically assisted anoplasty in addition to urethrorectal fistula closure; however, the blind end of the colon canal was not sent for postoperative pathological examination. On the seventh day after surgery, he had fully recovered and was released. Anal dilators are numbered from 7 to 13 after discharge and regular anal expansion once per day. Six months later, he returned to the hospital to have the transverse colostomy closed. The child starts to defecate on day, and the patient was discharged one week after surgery.

At the age of eight months, or two months after surgery, the infant developed abdominal distension, regurgitation, and additional symptoms. Radiographs of the abdomen in standing position revealed a low intestinal obstruction (Figure 1C). During the period, the patient continued to use the No. 15 anal dilatation implement daily to dilate the anus, and the child experienced daily exhaustion and defecation after dilation. Patients were discharged after gastrointestinal decompression, antibiotics, intestinal motility medications, and other conservative treatments alleviated their symptoms. However, after being discharged, the patient continued to experience diarrhea, vomiting, constipation, and other symptoms, and was referred to our hospital and local hospitals multiple times. The infant also experienced weight loss, losing 1.5 kg in three months.

At the age of 11 months, the child was referred to our hospital again for intestinal obstruction. Barium enema examination showed that the diameter of the rectum, sigmoid and descending colon tubes was small, with the narrowest diameter about 1.4 cm, and the transverse colon near the spleen showed the transitional segment, slightly flared shape, and the transition zone is in the splenic flexure of the colon, which the widest part was about 3.2 cm (Figure 1D), and 24 h postevacuation radiographs had no retention of barium.

**Figure 2:**
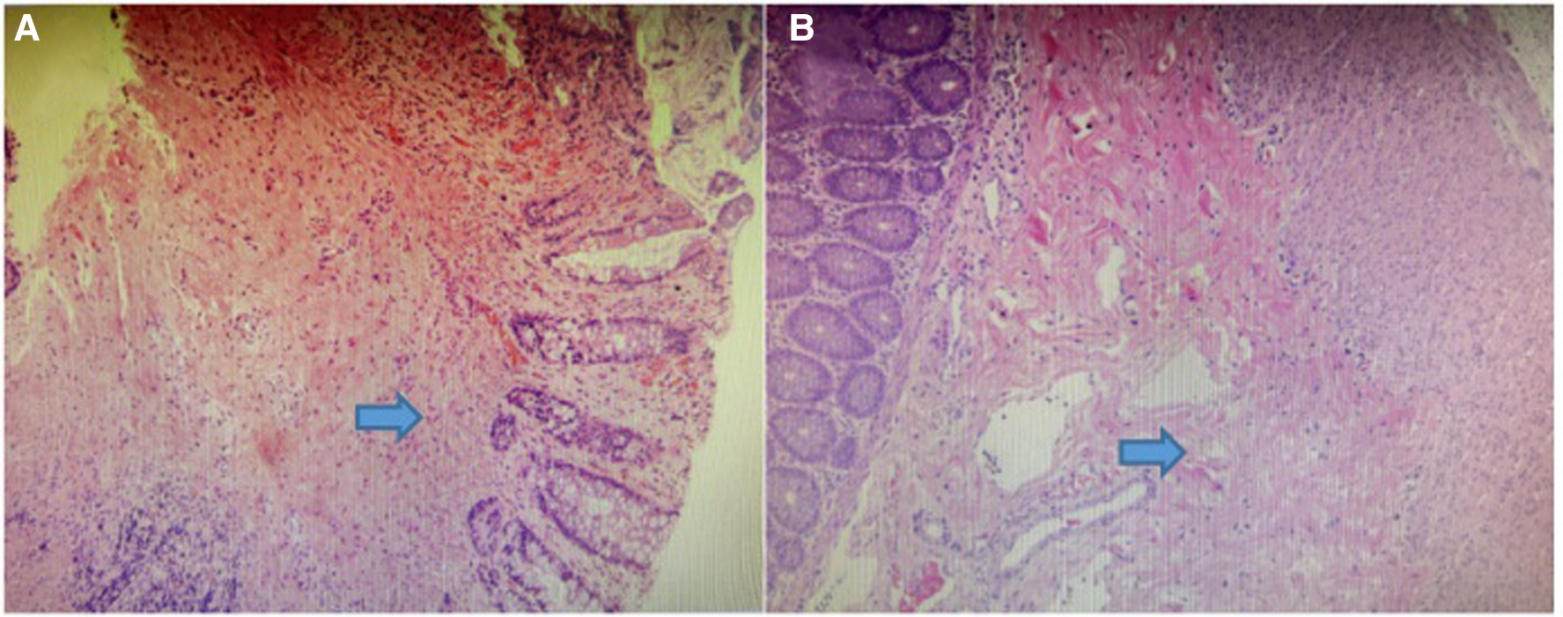
(**A**) photomicrograph showing absence of ganglion cells in submucosal plexus. (**B**) photomicrograph showing absence of ganglion cells in enteric intermuscular plexus.

**Figure 3:**
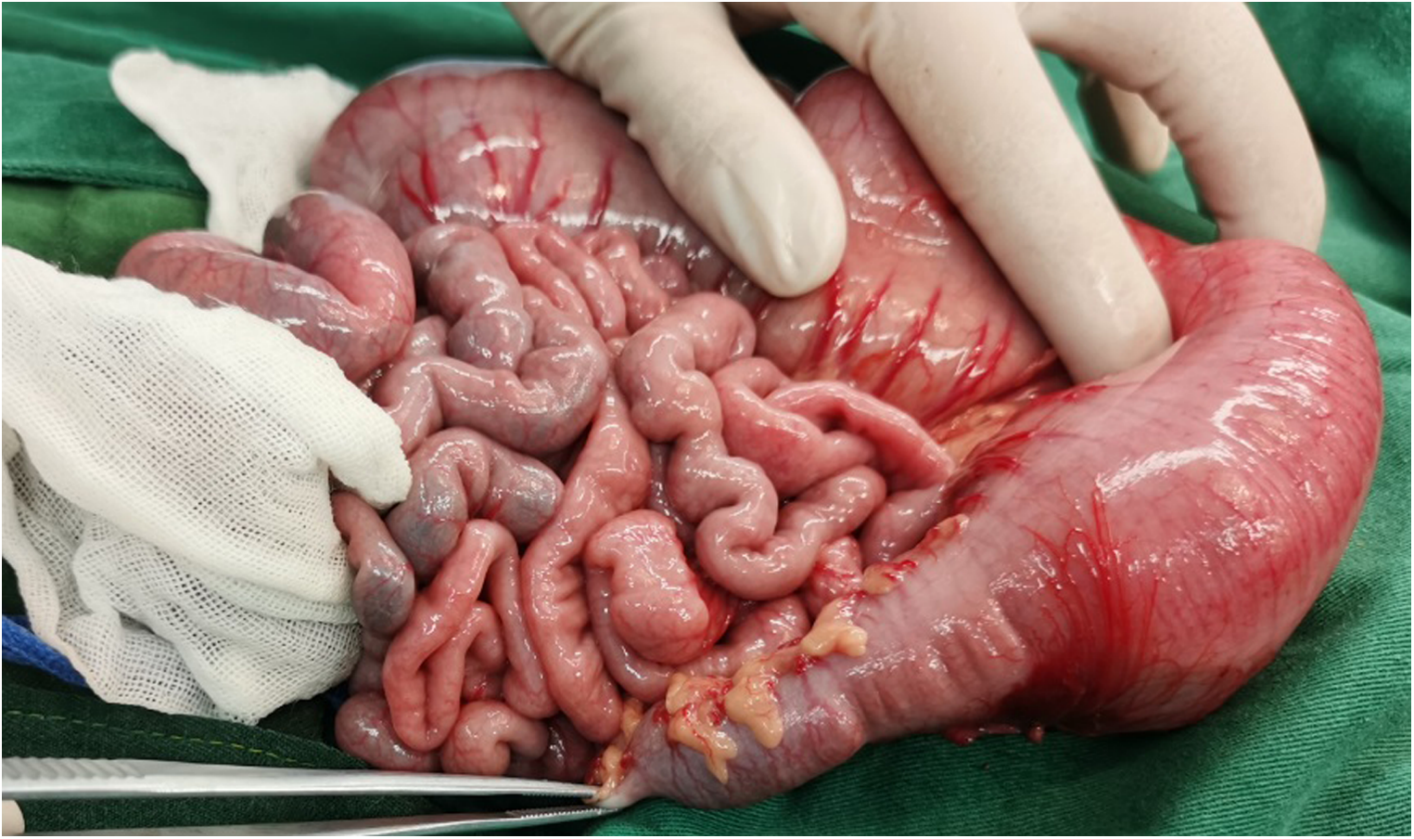
The rectum, sigmoid and descending colon were narrow that about 1 cm, the intestinal wall was thickened and pale, and the colon pocket was not obvious,and the proximal descending colon was gradually dilated, and the transverse colon and ascending colon were significantly dilated that about 6 cm.

The child's rectum was biopsied, and the pathological results revealed that neither the submucosal nor intermuscular nerve plexuses contained any ganglion cells (Figure 2). We diagnosed the patient with HSCR based on the patient's symptoms, barium enema examination, and the pathological results of the rectal biopsy. The no-aganglionic segment was long, extending to the transverse colon. The infant underwent a transverse colostomy and multiple intestinal biopsies due to the ineffectiveness of colon lavage. During this operation, it was determined that the rectum, sigmoid, and descending colon were narrow by approximately 1 cm, that the intestinal wall was thickened and pale, that the colon pocket was not visible, that the proximal descending colon was gradually dilated, and that the transverse colon and ascending colon were significantly dilated by approximately 6 cm (Figure 3). Intestinal biopsies were also obtained from the upper rectum, the middle of the descending colon, and the middle of the transverse colon. Pathological findings revealed that neither the upper rectum nor the middle descending colon contained ganglion cells, but the middle section of the transverse colon did.

The 18-month-old was brought back to the hospital to undergo a pull-through procedure. During the operation, a biopsied sample of the proximal intestinal tract of the transverse colostomy revealed nerve plexus and well-developed ganglion cells. Therefore, we lower the proximal intestinal conduit of the transverse colostomy to the anus. The patient began to eat one week after surgery and was discharged from the hospital after two weeks of excellent recovery.

Currently, after six months of follow-up, the patient still has enteritis, which can be treated with intestinal washing and oral antibiotics, as well as intermittent fecal contamination, and his weight has increased by 3 kg compared to before surgery.

## Discussion

While both ARM and HSCR are prevalent congenital malformations in newborns, the presence of ARM in conjunction with HSCR is extremely uncommon, with an incidence of only 2.4%–3.4% (6). Hirschsprung's disease and anorectal deformity currently have no identifiable cause. Some theories cover hereditary, familial, and environmental elements (7, 8).

HSCR usually shows up as delayed meconium discharge and abdominal distension in newborns. Children with medium-high ARM often need a diverting stoma. The position of the stoma above the absence of the ganglia does not produce the typical distended symptoms (9, 10). Due to defects in the development of the anal sphincter, pelvic floor muscle, and pelvic nerve, infants with ARM frequently experience postoperative defecation dysfunction, such as constipation and fecal fouling (11). As a consequence, HSCR ileus symptoms may be misinterpreted as postoperative complications, resulting in a missed diagnosis of potential comorbiditis. Two days after birth, a transverse colostomy was performed on the patient. Therefore, the patient had no symptoms of abdominal distension or progressive constipation prior to the conclusion of the ostomy reduction surgery, and the patient's meconium discharge could not be evaluated due to anal atresia.

Therefore we mistook the child's constipation, abdominal distension, and other symptoms for complications following ARM correction surgery. All of these factors ultimately cause us to delay the patient's diagnosis and treatment. After the patient repeatedly exhibited symptoms of enteritis and constipation, we realized that the patient was suffering from additional diseases. Barium enema examination and biopsies of the rectum or colon ultimately confirmed the diagnosis of HSCR.

**Figure 4:**
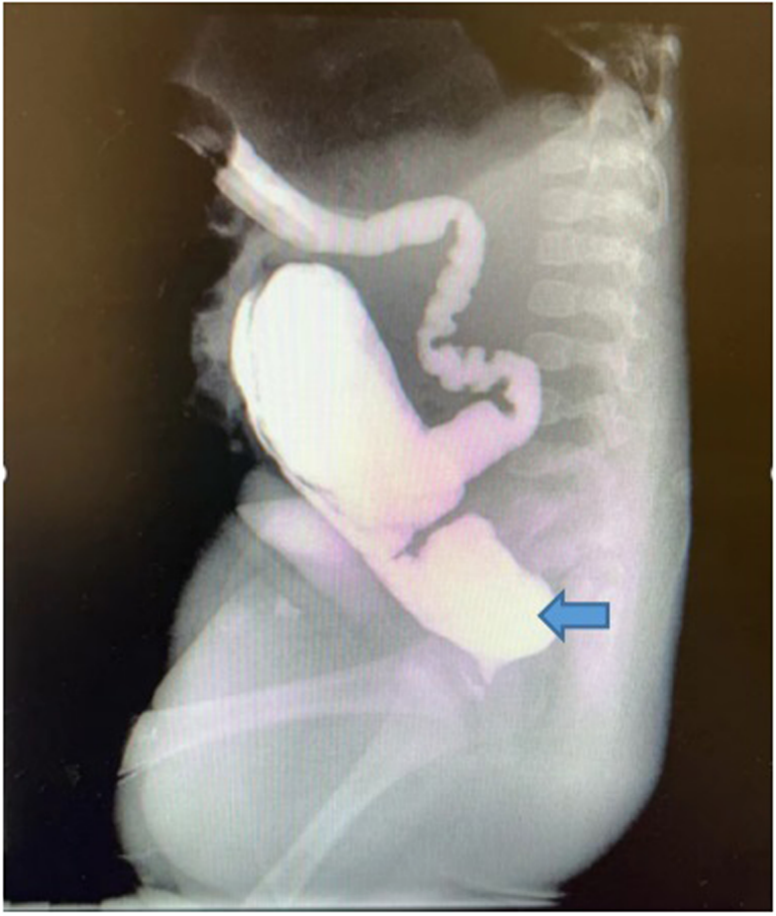
The blind end of the distal bowel is dilated in ARM children without Hirschsprung's disease. We took that picture of barium enema contrast before the anoplasty.

In reviewing this case, we discovered that the barium enema examination performed prior to the second phase ARM correction operation on this child revealed that the blind end of the colon was small and the haustrum was not obvious. All of those are different from those of typical ARM children, the majority of whom exhibit dilatation of the colon's blind end and significant colonic pocket (Figure 4).

Due to a incomplete barium enema examination reading, we lost the ideal opportunity to diagnose Hirschsprung's disease. In addition, when the child had enteritis and constipation, we did not conduct a barium enema examination in a timely manner. In the study by Watanatittan et al. (12), barium enema examination did not reveal a significant segment of stenosis and spasm. But during this examination, we discovered a significant stenosis and spasm segment, but the 24 h postevacuation radiographs showed no barium retention. In contrast to the typical barium examination for Hirschsprung's disease, the radiographs taken 24 h after evacuation showed barium retention. We believe this was due to the child's intestines experiencing acute stress.

The gold standard for diagnosing HSCR is the absence of ganglion cells in the distal intestinal intermuscular and submucosal nerve plexus (13, 14). There is a normal ganglion-free zone 1–2 cm above the dentate line in the human body, meaning that ganglion cells lack ganglion (i.e., in the anal transition zone, ATZ) (15). The ATZ is the middle portion of the anal canal where the squamous epithelium of the anus progressively transforms into mucosa of the colorectal type. Typically, ATZ extends upward from the dentate line between 0.6 and 2 cm. In patients with ARM, the ATZ structure is also typical of the distal fistula/rectum. If specimens are collected from these regions, ganglion cells may be visibly absent, producing false-positive results. Therefore, the patient was not sent for routine examination of the rectal terminal bowel tract, and we missed an opportunity to detect HSCR early.

## Conclusion

There is still the chance of HSCR-complicated ARM cases. Before an anoplasty, care must be given to the cologram. Attention should be paid to colography before anoplasty. Its abnormal morphology can indicate the possibility of HSCR in children with ARM. For patients with intractable constipation and recurrent enteritis, the possibility of HSCR should be suspected, and rectal biopsy should be performed to confirm the diagnosis if necessary.

## Data Availability

The original contributions presented in the study are included in the article, further inquiries can be directed to the corresponding author.
